# Biomaterials as Haemostatic Agents in Cardiovascular Surgery: Review of Current Situation and Future Trends

**DOI:** 10.3390/polym14061189

**Published:** 2022-03-16

**Authors:** Horațiu Moldovan, Iulian Antoniac, Daniela Gheorghiță, Maria Sabina Safta, Silvia Preda, Marian Broască, Elisabeta Badilă, Oana Fronea, Alexandru Scafa-Udrişte, Mihai Cacoveanu, Adrian Molnar, Victor Sebastian Costache, Ondin Zaharia

**Affiliations:** 1 Department of Cardiovascular Surgery, Bucharest Clinical Emergency Hospital, 014461 Bucharest, Romania; mariasabinasafta@gmail.com (M.S.S.); dr.silvia.preda@gmail.com (S.P.); marian.broasca@gmail.com (M.B.); alexscafa@yahoo.com (A.S.-U.); catalin.cacoveanu@gmail.com (M.C.); 2 Faculty of Medicine, Carol Davila University of Medicine and Pharmacy, 050474 Bucharest, Romania; elisabeta.badila@gmail.com (E.B.); dr.fronea79@gmail.com (O.F.); ondin.zaharia@gmail.com (O.Z.); 3 Faculty of Materials Science and Engineering, Politehnica University of Bucharest, 060042 Bucharest, Romania; antoniac.iulian@gmail.com; 4 Faculty of Medicine, Iuliu Hateganu University of Medicine and Pharmacy, 400000 Cluj-Napoca, Romania; adrianmolnar097@gmail.com; 5 Heart Institute, 400001 Cluj-Napoca, Romania; 6 Sf. Constantin Hospital, 500388 Brasov, Romania; victorscostache@gmail.com; 7 Faculty of Medicine, Titu Maiorescu University, 040441 Bucharest, Romania; 8 Prof.Dr. Theodor Burghele Clinical Hospital, 050659 Bucharest, Romania

**Keywords:** haemostasis, cardiovascular surgery, coagulation, haemostatic agents, biomaterials

## Abstract

Intraoperative haemostasis is of paramount importance in the practice of cardiovascular surgery. Over the past 70 years, topical haemostatic methods have advanced significantly and today we deal with various haemostatic agents with different properties and different mechanisms of action. The particularity of coagulation mechanisms after extracorporeal circulation, has encouraged the introduction of new types of topic agents to achieve haemostasis, where conventional methods prove their limits. These products have an important role in cardiac, as well as in vascular, surgery, mainly in major vascular procedures, like aortic dissections and aortic aneurysms. This article presents those agents used for topical application and the mechanism of haemostasis and offers general recommendations for their use in the operating room.

## 1. Introduction

Intraoperative and postoperative haemorrhage is a relatively common event during and after open heart procedures, performed with extracorporeal circulation and various degrees of hypothermia. The average incidence is 5–9%, with limits between 0–16%, of all unselected cardiac surgical patients [1,2]. The control of intraoperative and postoperative bleeding is essential in the practice of cardiovascular surgery because the reopening of the surgical wound and transfusion of blood products are both associated with poor outcomes. The reoperated patients have approximately three-times greater mortality risk than non-reoperated. Surgical causes of postoperative bleeding are found in 66% of cases on re-exploration, only 33% being attributed to various types of coagulation disorders. Surgical bleeding is considered to have its origin on important vessels, surgical suture lines, or anastomosis, whereas nonsurgical bleeding originates in small vessels and tissue surface, being the result of a coagulation disorder [3,4]. Haemostatic agents play a key role in establishing haemostasis and preventing haemorrhage-associated death.

Blood transfusions occur in approximately 21% of all operations, and in 45.8% of cardiac cases, however, they are associated with multiple risks and complications. Blood transfusion is also associated with immunomodulation, bacterial infection, and a multitude of non-infectious, but important, complications [5,6,7].

Haemostasis should be achieved by systemic and local methods.

Systemic administration of blood products and procoagulant medication is of great importance, but they also carry large risks. Systemic agents, such as antifibrinolytics and recombinant Factor VII, are generally used to counter coagulation but they are expensive. There is not strong evidence to support the use of recombinant Factor VII in most cardiovascular operations [8,9].

The first local methods of direct surgical control of suturing lines, anastomosis, and other points of the surgical field, are classical surgical procedures, like suture applications and vessel ligation, or electrocautery. There are many haemostatic manoeuvres, from simple digital pressure application, electrical tissue cauterization, and warm saline irrigation of the wound to topical application of procoagulant agents [10].

Those novel adjuncts that have evolved over decades are effective towards achieving haemostasis during cardiovascular procedures. They include topical gelatines, collagens, oxidized celluloses, thrombin and fibrin sealants, synthetic glues, and glutaraldehyde-based glues. The effective use of topical agents is highly dependent on the surgeon’s experience or preference and their availability in the surgical setting. Local treatments also carry risks, and their efficiency has not been extensively studied in large randomized, placebo-controlled prospective studies. The relatively recent expansion of minimally invasive techniques in cardiac surgery has led to an increasingly routine use of these novel agents in order to achieve haemostasis where conventional suturing has proven difficult [10].

In addition, the widespread use of antiplatelet therapy, such as aspirin and clopidogrel in the management of coronary heart disease, is likely to increase the challenge of haemostasis, particularly in emergent patients [11].

The cardiovascular surgeon is now faced with an ever-increasing array of haemostatic agents, each armed with subtly different qualities and proven in different contexts with various levels of evidence. In this article, we review the aetiology of bleeding in cardiovascular surgery and the coagulation cascade. We will describe the existing haemostatic topic agents and their application techniques [12].

## 2. Specific Aspects Related to Bleeding in Cardiovascular Surgery

Bleeding after extracorporeal circulation is often multifactorial, can be related to intrinsic haemostatic abnormality, excessive anticoagulation and imperfect reversal, or platelet disfunction [4]. Haemostasis consists of complex interactions between the extracorporeal circuit, damaged endothelial and subendothelial cells, platelets, leucocytes, coagulation factors and coagulation inhibitors like heparin, which eventually results in the polymerisation of fibrine monomers to form a stable clot [13]. Disfunctions in any of these components can result in deficient intraoperative haemostasis. An important role is the effect of prolonged extracorporeal circulation and hypothermia. Understanding the coagulation cascade and the mechanism of action of different haemostats will aid the surgeon in choosing the most appropriate haemostatic agent and strategy for each patient [1].

**Figure 1:**
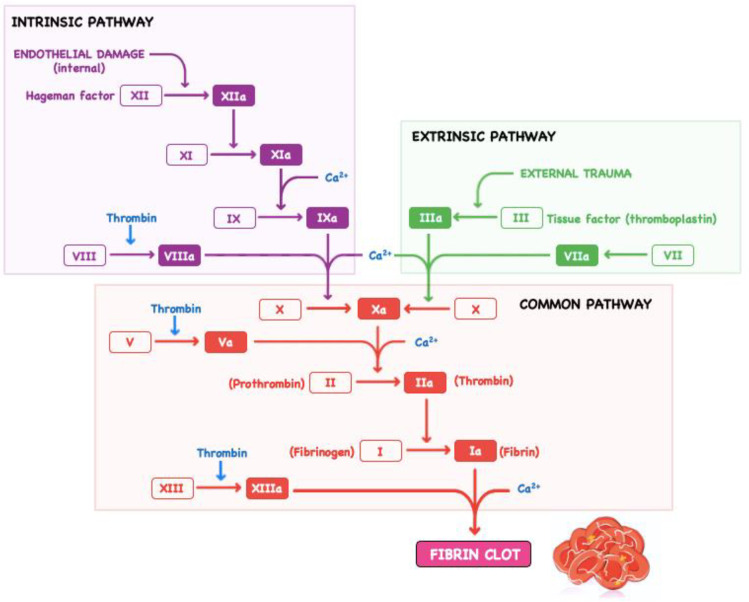
Schematic representation of coagulation cascade.

Contact between blood and the artificial surface of the extracorporeal circuit and cardiac or vessel wall injury is the trigger for the initiation of the coagulation cascade (Figure 1). This activates endothelial cells and platelets to mobilize adhesion molecules to the cell surface, which, in turn, promotes aggregation of platelets, leucocytes, and monocytes on the surface of the extracorporeal circuit and activated endothelial cells, thereby assisting in localising the site of thrombus formation [14,15]. Activated endothelial cells and platelets also release the von Willebrand factor, which binds the platelets to extravascular structures, like pericardium and subendothelial connective tissue, and promotes further platelet aggregation [13]. The formed leucocyte–platelet aggregate provides a secure surface for the subsequent formation of a stable fibrin clot in the ensuing process of the coagulation cascade [15]. Tissue factor is released by injured cells and initiates a cascade of coagulation factor activation (extrinsic pathway) that drives the production of thrombin. Small amounts of thrombin are produced initially [15]. The thrombin produced further activates platelets and triggers a separate way of coagulation factors (intrinsic pathway), which results in the production of factor IXa and VIIIa complexes [16,17]. In the presence of calcium ions, this complex catalyses the activation of factor X and amplifies thrombin production [18]. Thrombin works by catalysing the conversion of fibrinogen to fibrin, activating factor XIII to stabilize the clot, and activating the fibrinolysis inhibitor.

Cardiovascular surgery is generally associated with perioperative blood loss and a high risk of perioperative blood transfusion. Bleeding can have significant clinical and economic impact, including increased health care utilization and poorer patient outcomes [19]. The wide range of cardiopulmonary bypass application distinguishes this discipline from other surgical specialities. The evolution of haemostasis and perioperative care from the early days of cardiovascular surgery to the modern era offers a large perspective on the developments of surgical techniques, transfusion, and cardiopulmonary by-pass technology.

The relatively recent expansion of minimally invasive approaches in cardiac surgery have led to an increasingly routine use of these topical agents in order to achieve haemostasis where conventional suturing and other techniques have proven difficult. In addition, the widespread use of antiplatelet therapy, such as aspirin and clopidogrel, in the treatment of cardiovascular patients is likely to have increased the challenge of haemostasis, particularly in emergent patients.

Surgeons today are faced with an increasing array of haemostatic agents, each with different qualities proven in different contexts with various levels of evidence. Most times, the choice of haemostatic agents is made without an in-depth knowledge of the agent’s composition, mechanism of action, or efficacy.

The circumstances surrounding the desire to use a haemostatic agent are crucial to the choice of agent. Haemostatic agents that accelerate clot formation have the disadvantage of only being effective against surgical bleeding when haemorrhage is minimal. This relegates the agents to either a prophylactic role in which the surgeon must always anticipate excess haemorrhage, during cardiopulmonary bypass or after anticoagulation reversal with protamine.

The alternative option favoured by some surgeons is to use the bovine serum albumin and glutaraldehyde tissue adhesive with its ability to rapidly form a mechanical seal that is independent of native clotting factors and which forms even in the presence of some moderate bleeding. Selection of haemostatic agents to promote intraoperative haemostasis have been published but the scarcity of literature makes development of detailed guidelines difficult [20,21].

After achieving surgical haemostasis, it is mandatory to deploy one of the compression haemostatic agents, such as oxidized regenerated cellulose, microporous polysaccharide hemispheres, or microfibrillar collagens. These offer the advantages of reducing active bleeding. The haemostatic adjuncts, such as gelatine–thrombin matrix sealant (Floseal), are useful when bleeding is not amenable to standard techniques. The use of those products is associated with side effects, such as immunologic reactions, antibody development, and transmission of viral infections, particularly in active and fibrin sealants, and, potentially, increased mortality [22]. But there are important benefits to using them. Active and flowable haemostatic products have been shown to produce higher haemostatic success rates compared with mechanical agents. Fibrin sealants have also shown efficacy compared with conventional haemostasis and other mechanical agents [23,24,25].

**Figure 2:**
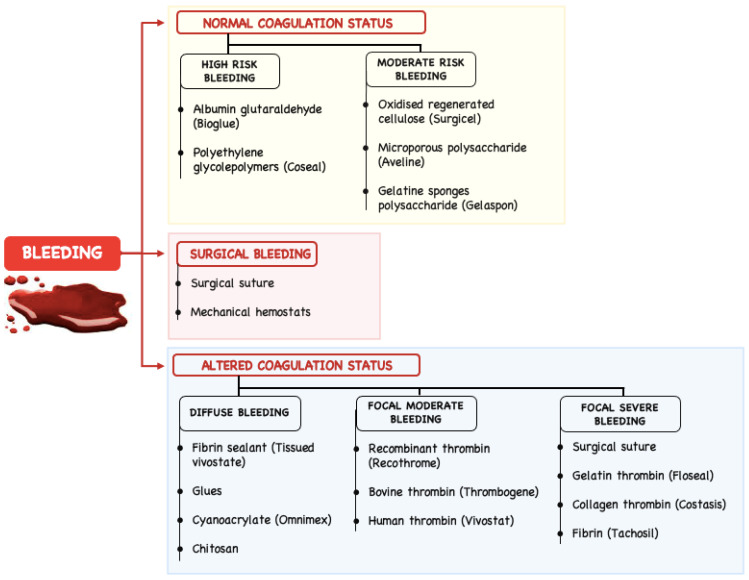
Bleeding management using haemostatic agents.

The albumin and glutaraldehyde combination (Bio Glue) include the reported risks of embolization, local tissue destruction, and the bovine source of the albumin with the related risks or immunologic effects but they are useful in major aortic surgery, as aortic dissection or thoracic aneurysms. The surgeon should evaluate when is appropriate to use each haemostatic agent and when the benefit outweighs the risk, such as in cases where haemostasis cannot be achieved with mechanical means and systemic administration of blood products or other procoagulant medication. There are preliminary clinical positive results, but there is a need for further randomised control trials comparing different topical local haemostatic adjuncts used in cardiovascular surgery, given the important problem of bleeding side effects [26,27]. Figure 2 presents a scheme representing the bleeding management using haemostatic agents.

## 3. Current Haemostatic Agents and Their Clinical Use in Cardiovascular Surgery

In the following sections we present the classical and novel haemostatic agents used in cardiovascular surgery [20,21]. A classification based on the component used of available commercial haemostatic agents is presented in Table 1.

### 3.1. Conventional Haemostatics in Cardiovascular Surgery

Bleeding from cardiac or vessel structures can easily be controlled using conventional methods, such as simple sutures, pledged sutures, compression with cottoned patties, electrocautery, or use of vascular ligatures and clips [5]. Diffuse capillary bleeding may further require warm saline irrigation or the use of topic haemostatic adjuncts.

Direct placement of sutures, ligature, vascular clips, or even only simple digital pressure can provide haemostasis. But small-vessel or capillary bleeding require different methods.

Electrocautery often achieves haemostasis adequately. This method can place adjacent suture lines or anastomoses at risk of thermal injuries and dramatic haemorrhagic consequences. Furthermore, it can cause complete vessel lumen occlusion with subsequent compromise of the perfused territory [28].

Warm saline irrigation is often used in cardiac surgery to achieve haemostasis [29]. Its mechanism of action consists of the mechanical compression from the oedema of surrounding tissues and the vasodilatation of bleeding vessels that reduce intraluminal pressure. Also, the washing effect of warm saline can stop a local fibrinolytic process.

Bone wax, consisting of bees’ wax and a softening agent, such as paraffin or Vaseline, is commonly used to stop bleeding resulting from the sternum by physically obstructing the spongiosa of the bone. Unfortunately, it can cause side effects, like osteomyelitis, granuloma, or imperfect sternum healing [28].

### 3.2. Haemostatic Agents with Active Mechanisms in Cardiovascular Surgery

Understanding of the coagulation cascade and the means to produce coagulation factors safely and on a large scale has led to the introduction of novel haemostatic agents that work independently of the coagulation mechanisms [30,31]. These agents introduce extrinsic clotting factors on application and mimic various stages of the coagulation process.

**Figure 3:**
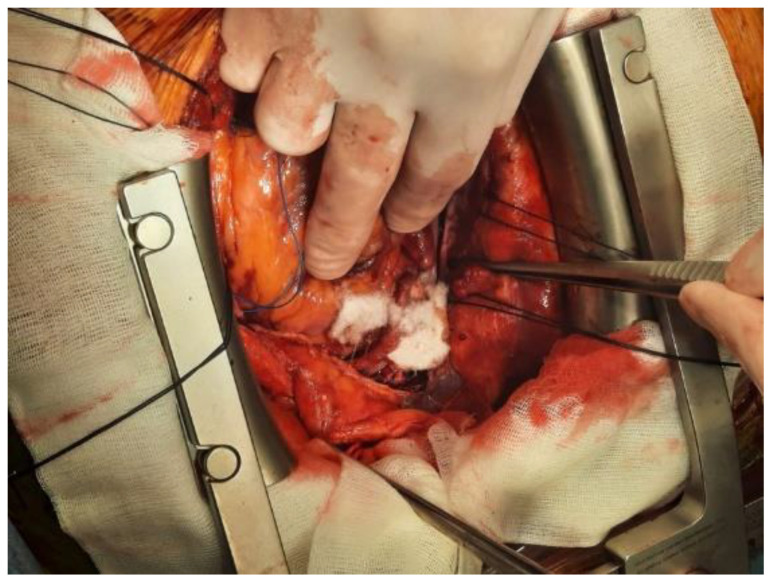
Intraoperative aspect of bleeding management using TachoSill.

The principal products of this category include thrombin sealants (Thrombin, Thrombogen, and Thrombistat), fibrin sealants (Tissel), fibrin patches (TachoSill, and TachoComb), autologous fibrine and thrombocytes concentrates (VivoStat) and gelatine–thrombin matrix sealant (FloSeal). Figure 3 presents the intraoperative use of TachoSill.

#### 3.2.1. Thrombin Sealants

It is the most used adjunctive haemostat in general surgical procedures in the United States. Thrombin is a serum protease that cleaves fibrinogen into fibrin and activates factor XIII generating the stabilization of the clot. Generally, the thrombin sealants used in cardiovascular surgery are derived from bovine plasma. From January 2008, recombinant human thrombin (Recothrom (ZymoGenetics, Seattle, WA, USA)) gained United States Food and Drug Administration approval [32,33].

#### 3.2.2. Fibrin Sealants and Fibrin Patches

Fibrin sealants and fibrin patches (Tisseel, Beriplast, Hemaseel, Crosseal/Quixil, and Vivostat) have been introduced for the first time in clinical practice by Matras and co-workers [34]. They prove to have an important role within various types of interventions in cardiovascular surgery, redo cardiac procedures, and major aortic procedures, in treating or preventing diffuse bleeding [34,35].

Fibrin sealants are made of two components contained in separate vials: a freeze-dried concentrate of fibrinogen, factor XIII, and fibronectin (the sealant) and a freeze-dried thrombin (the hardener).

Fibrinogen is the precursor of fibrin, the strong element of the clot. The transformation of fibrinogen into stable fibrin occurs by means of thrombin and factor XIII, which in turn are activated by thrombin. Fibrin sealants reproduce the last step of the coagulation cascade. They have the advantage of being independent from the patient’s clotting mechanism, and they are also effective in patients with coagulation disturbances after extracorporeal circulation or those who are receiving antiaggregant or anticoagulants [36,37]. Fibrin sealants contain bovine aprotinin or tranexamic acid as antifibrinolytic agents to slow fibrinolysis at the application site. There are reports of the adverse effects of bovine aprotinin in sealants relating to the highly immunogenic nature of bovine aprotinin in humans. About 125 anaphylactic reactions are reported in the literature, with an estimated anaphylaxis incidence between 0.5 and 5 per 100,000 topical applications [38].

Vivostat (Vivostat A/S, Birkerod, Denmark) is a system that produces an autologous fibrin sealant. The device can generate 4.5 mL of sealant from 120 mL of the patient’s blood in 23 min. This is done before surgery. Application of the agent can be done using a spray system. There is no randomized controlled trial using this system in cardiac surgery where it is compared with any other sealing agents, although its tensile strength has been compared with other fibrin sealants and has been found comparable. There are 24 trials reported in the literature of the use of fibrin glues in cardiothoracic surgery, with 20 trials reporting a positive benefit in terms of reducing bleeding, 4 studies reporting no difference from controls, and no studies reporting a negative influence on bleeding [38,39].

Codispoti and Mankad published a randomized controlled trial in pediatric cardiac surgery in 2002. They have shown a significant reduction in the use of blood products and time to achieve haemostasis when fibrin sealant has been used in in cases with proven coagulopathy [40]. There are reports of an increased risk of myocardial injury or even death in coronary artery bypass grafting patients when fibrin sealant was applied on anastomoses. The patients have had an acute occlusion of bypass grafts and immediate embolectomy showing fresh fibrin clot in the lumen of the grafts in each case. This study specifically emphasizes that using fibrine sealant in coronary operations must be avoided [41].

### 3.3. Haemostatic Agents with Nonactive Mechanisms in Cardiovascular Surgery

They are nonactive products and they do not contain clotting factors. They include mechanical haemostatic adjuncts and synthetic sealants. Conventional topic haemostatic adjuncts work by forming physical lattices that promote clot formation [12].

#### 3.3.1. Gelatine Sponges

Like Gelfoam (Baxter, Hayward, CA, USA), Gelaspon, whose efficacy relies on a porous consistency to absorb large quantities of blood, the weight of which subsequently tamponades the underlying bleeding surface [42,43].

#### 3.3.2. Microfibrillar Collagens

Like Avitene Flour MCH (Davcol, Warwick, RI, USA), Colgel, and Helitene, promote platelet aggregation and adhesion without direct effect on the soluble components of the clotting system [28]. This agent was first described in 1969. It is a water-insoluble acid salt of bovine collagen, which, when used topically, adheres to the tissue and provides some haemostatic effect by reinforcing the fibrin clot. For redo cardiac operations, major vascular procedures, such as ascending aortic aneurysm repair or aortic dissection necessitating deep hypothermic circulatory arrest, or without deep hypothermic circulatory arrest, microfibrillar collagen has the ability to reduce postoperative bleeding and was found to be useful in reducing postoperative drainage [44]. Microfibrillar collagen is reported to cause end-organ damage if returned to the circulation by either pump suction or cell salvage devices [45].

#### 3.3.3. Oxidised Regenerated Cellulose

**Figure 4:**
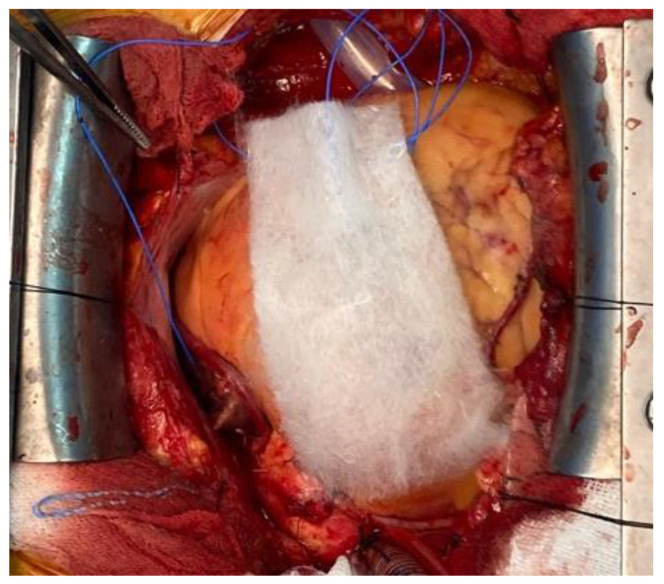
Intraoperative aspect of Fibrilar oxidised cellulose.

Like Surgicel (Ethicon, Somerville), this has an acidic quality that enables reaction with blood to precipitate an artificial coagulum that provides a scaffold on which platelets can start the adhesion and aggregation process leading to coagulation, but not interfering the physiological clotting mechanism [46]. Oxidised regenerated cellulose is a polyanion, the functional unit of which is polyanhydroglucuronic acid. After surgical application there is evidence of absorption within the first few days, and complete absorption can be observed the next 4 to 8 weeks [47]. This rate being dependent on the volume of material used, local vascular activity, and the histological structure of the tissue on which is applied. Surgicel has bacteriostatic capacity with reported antimicrobial activity, even against antibiotic-resistant micro-organisms [48,49]. Figure 4 presents the intraoperative aspect of Fibrillar oxidised cellulose.

#### 3.3.4. Microporous Polysaccharide Hemispheres

Like Arista (Medafor, Minneapolis, MN, USA), by absorbing fluid and small molecular blood components, this generates a scaffold that concentrates platelets and coagulation proteins on its surface and, thereby, activates the extrinsic clotting pathway [34]. Furthermore, this scaffold absorbs water, concentrating proteins and platelets locally at the wound’s edge [34]. During clot formation, they should be left in place, and then removed delicately to prevent clot elevation [50,51]. Because they do not contain specific coagulation factors, these mechanical topic haemostatic adjuncts must be used in patients who have a relatively normal coagulation system, in cardiac procedures, after the protamine administration and reversal of the heparin effect [26,27]. They are not efficient in coagulation disturbances or/and thrombocytopenia. However, many of these conventional local haemostatic adjuncts can be soaked in thrombin prior to use for enhanced haemostasis [32].

### 3.4. Haemostatic Agents with Active and Nonactive Mechanisms

There are mechanical haemostatic agents with thrombin, also known as flowable agents. They combine mechanical and active properties and work by promoting thrombus formation at the bleeding site [27,52].

#### 3.4.1. Gelatine–Thrombin Matrix Sealant

Like Floseal, this was first introduced in neurosurgery in 1999 and after, in cardiovascular procedures [32]. FloSeal Haemostatic Matrix (Baxter Healthcare Corp, Freemont, CA, USA) is a combination of a bovine gelatine scaffold and a human thrombin. In contact with fluids in the surgical wound, the gelatine particles swell and tamponade the bleeding, and the high thrombin concentration accelerates clot formation. The thrombin used has a significantly reduce viral load, but no procedure has been shown to be completely effective in removing viral infectivity from derivatives of human biological products. Absorption of FloSeal starts in the first 2–4 days and is complete after 6–8 weeks. FloSeal is superior in achieving haemostasis before reversal of the heparin effect with protamine [53,54].

#### 3.4.2. Composite Collagen and Thrombin Sealant

Like CoStasis (Cohesion Technologies, Palo Alto, CA, USA), this agent is a composite of bovine microfibrillar collagen and bovine thrombin in a calcium chloride buffer. This composite is mixed during the surgical procedure with an equal volume of autologous plasma. The patient’s plasma provides fibrinogen that is fractionated by thrombin to form a collagen–fibrin scaffold. The agent is used as a spray over the surgical field. CoStasis has been shown to achieve haemostasis within 3 min in cardiac surgery patients undergoing or coronary artery bypass grafting. But has no impact on reducing transfusion of blood products [55]. Unfortunately, bovine thrombin preparation can induce the development of bovine thrombin associated factor V antibodies, which may lead also to coagulation disturbances [56]. The risk of acquired factor V antibodies has declined due to the use of recombinant human forms of thrombin [54].

### 3.5. Tissue Sealants

Tissue sealants carry no intrinsic haemostatic factors, but, instead, act as physical barriers at the bleeding site.

#### 3.5.1. Absorbable Cyanoacrylate

**Figure 5:**
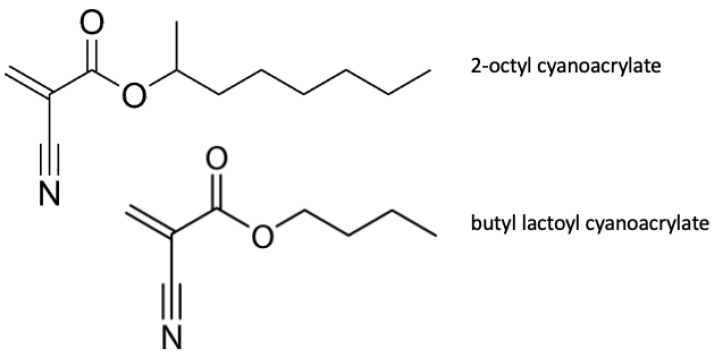
Chemical formulas of 2-octyl cyanoacrylate and butyl lactoyl cyanoacrylate.

Like OMNEX (Ethicon), this is a polymer that adheres to tissue. This agent forms a seal adherent to both synthetic material and human tissue that acts as a physical barrier but does not induce clot formation. The substance is a synthetic adhesive consisting of a blend of two monomers (2-octyl cyanoacrylate and butyl lactoyl cyanoacrylate). The chemical formulas of the monomers are presented in Figure 5. The seal degrades with time, breaking down into smaller absorbable fragments.

Lumsden and Heyman published in 2006 a prospective, randomized controlled trial for assessing the efficacy of cyano–acrylate sealants in establishing haemostasis of expanded polytetrafluoroethylene to arterial vascular anastomoses in arteriovenous grafts and femoral bypass grafts. They showed that haemostasis was achieved in 54.5% of patients receiving cyanoacrylate surgical sealant and in 10% of those receiving the control [57].

#### 3.5.2. Glutaraldehyde/Bovine Albumin

Like BioGlue (Cryolife, Kennesaw, GA, USA), this is a tissue glue that polymerises immediately and establishes a firm adhesive bond [31,58]. Two components are dispensed by a double-chambered syringe. The components are mixed within the applicator tip where polymerisation begins. The glutaraldehyde molecules bond the albumin molecules to each other and the tissue proteins at application site, generating a mechanical seal. This process is totally independent from the coagulation mechanism [52]. BioGlue begins to polymerize rapidly in 20 to 30 s and reaches its maximum strength in 2–3 min. BioGlue also adheres to synthetic graft materials through mechanical interlocks within the interstices of the graft matrix.

Coselli and co-workers showed in a randomized controlled trial that anastomotic bleeding and pledged use was significantly reduced in the BioGlue group compared with the control group [59]. The product has as side effects nerve tissue and conduction tissue injury generating AV block, [60] leaking of the BioGlue through needle holes [61], and impaired aortic growth causing anastomotic stenosis [62]. Therefore, BioGlue is not recommended in pediatric surgery, and caution must be taken in the application on aortic suture line. Disfunction of valve prosthesis has been also reported after application [63,64]. There have been reports regarding the potentially harmful effects of BioGlue to local tissues [65].

#### 3.5.3. Chitin and Chitosan-Based Haemostatic Agents (HemCon, Closure, Chitoseal, Celox)

Chitin polymer (-(1–4)-N-acetyl-D-glucosamine) is a polysaccharide synthesised by arthropods and fungi as a crystalline microfibril. Its use in cardiovascular surgery was first reported in 1983. It was shown to form a coagulum when in contact with heparinized and defibrinated blood [66].

**Figure 6:**
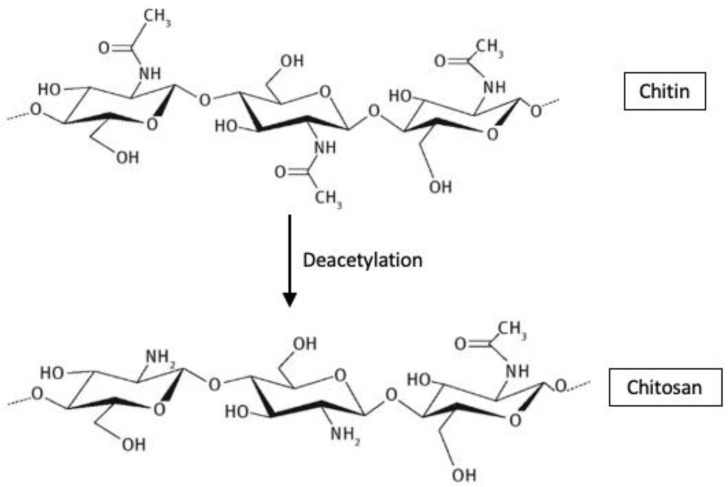
Chemical structures of chitin and chitosan.

Chitosan is a semisynthetic biomaterial obtained by deacetylation of chitin. Reports have indicated also that chitin and chitosan can play a role in the wound healing process [67]. The haemostatic mechanism of chitosan is independent of the classical coagulation cascade and seems to be an interaction between the substance and the cell membrane of erythrocytes [68,69]. The chemical structures of chitin and chitosan are shown in Figure 6.

#### 3.5.4. Polyethylene Glycol Polymers (CoSeal)

Polyethylene glycol polymers are synthetic hydrogels with application in cardiovascular surgery as tissue sealants. Those polymers propagate cross linking with various protein molecules, such as collagen, to form a cohesive scaffold that adheres to the tissue. The sealing capability of the hydrogel is almost immediate and does not require any human blood products or bovine components to inhibit bleeding. The suture lines during surgical placement of prosthetic vascular grafts treated with CoSeal achieved immediate haemostasis after re-establishment of blood flow [70,71].

## 4. Current Novelties in Research on Biomaterials for Haemostatics

### 4.1. Literature Overview Regarding Nanotechnologies and Novel Biomaterial for Haemostatics

Due to its unique advantages, nanotechnology has been receiving an increased interest and contributed to the development of haemostatic materials. Research has been conducted on the effects of different nanoparticles on the blood cells. Owing to their biocompatibility, high surface modification, and bonding capacity of the functional groups, polymeric nanoparticles seem to be promising as haemostatic agents.

Biomaterials that can facilitate the haemostatic and wound healing processes have been studied and developed continuously in the last decades. This perpetual research led to identification of new biomaterials, active components, and technologies that enhance haemostasis and promote tissue regeneration. A future trend is designing smart biomaterials that have the ability to mimic the native haemostatic processes.

Chitosan, a N-deacetylated form of chitin, is a promising biopolymer that exhibits high biocompatibility and haemostatic properties. Chitosan-based agents are very efficient in coagulopathy cases comparing to other commercial agents due to their independent haemostatic mechanism of the patient’s coagulation pathway [72].

Wang et al. [73] investigated the efficacies of chitosan-based gauze dressings and reported a significantly shortened time of haemostasis. Moreover, the chitosan dressing used in the study enhanced blood absorption, prolonged partial thromboplastin time and reduced antithrombin production. In a study performed by Gu et al. [74], chitosan was reported as better implantable haemostatic than gelatin sponge or oxidized cellulose related to the greater ability of chitosan to enhance platelet activation, morphological alteration, thrombin generation, and erythrocyte aggregation. In addition, Leonhardt et al. [75] obtained nanoscale chitosan mats and showed an increase interaction of chitosan with coagulation factors and platelets compared to Surgicel. These results indicate the effectiveness of chitosan as a haemostatic dressing.

In another study, Deprés-Tremblay et al. [76] reported that while interacting with the blood separation product—PRP (platelet-rich plasma), chitosan inhibits platelet-mediated clot retraction, increases the release of platelet growth factor, the duration of action and the bioactivity of PRP in vivo, which causes not only stable haemostasis, but also a long and effective antimicrobial and proregenerative effect.

Hangge et al. [77] applied nanotechnology for haemostats, reviewing the haemostatic performance of nanoparticles as topical and intravenous agents. Wang et al. [78] reviewed the developments in nanotechnology for both internal and external haemostatic agents, discussing the main problems of haemostatic nanomaterials. The research of Lashof-Sullivan et al. [79] on animals suffering from haemorrhage proved that the haemostatic nanoparticles used improved survival to 90% compared to 60% with no treatment. Biranje et al. [80] made a porous chitosan nanoparticle dressing and showed increased pro-coagulant activity through enhanced thrombin generation and controlled biodegradation and biocompatibility.

Future studies should focus on identification of new active components, modularizing haemostatic imitation, and improving mechanisms of various biomaterials and technologies to promote and strengthen active platelet aggregation in bleeding sites.

### 4.2. Collagen Loaded with Gelatin Microspheres as a New Composite Biomaterial for Haemostatics

Potential new biomaterials for haemostatics could be the collagen loaded with gelatin microspheres in different concentration: 4%, 21%, and 41%. In order to characterize these new biomaterials for haemostatics and observe the interface between collagen matrix and gelatin microspheres, different microscopical techniques were used: Scanning Electron Microscopy (SEM) and Atomic Force Microscopy (AFM).

SEM has been performed on a Philips XL 30 ESEM-TMP scanning electron microscope at 25 kV. The imaging details have been obtained using a combination of secondary and backscattered electrons. The samples have been directly mounted on the SEM sample stub without being sputtered with a conductive layer. The measurement has been performed at a vapor pressure of 0.7 Torr.

AFM studies have been performed on atomic force microscope type Veeco Multimode. In order to not damage the surfaces and to reduce shear forces, the AFM system works in “tapping mode”. Before being placed in the microscope, the samples were cut square pieces of 5 mm and fixed on the sample holder. Scanning parameters were: scan rate: 0.3 Hz; scan size: between 1 and 10 µm; scan angle: 90°; and line samples: 1024 (maximum value for a best resolution).

**Figure 7:**
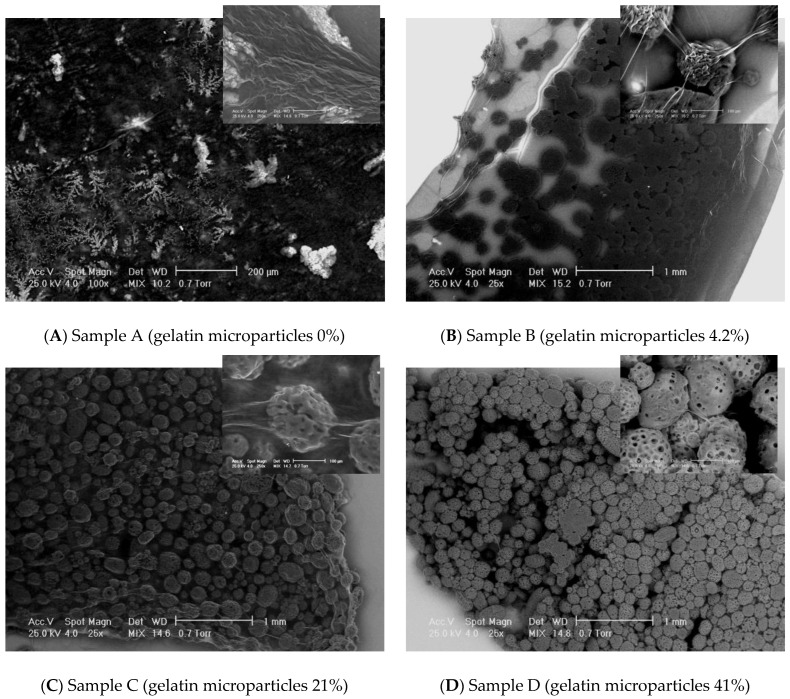
SEM results on the experimental samples collagen based loaded with gelatin microspheres ((**A**) sample A—0% gelatin, (**B**) sample B—4% gelatin, (**C**) sample C—21% gelatin, and (**D**) sample D—41% gelatin): general view (magnification 25×) and the interface aspects between collagen fibrils and gelatin microspheres (magnification 250×).

SEM images have been recorded at different magnifications to assess the general aspect of the samples surface morphology as well as to investigate the interface between the microparticles and the matrix. A general view on the samples’ appearance has been obtained through SEM micrographs taken at two magnifications: 25× (Figure 7). Non-loaded Collagen (sample A) has been used as control sample. It appears as a matrix containing collagen fibrils and some salt crystals. Increasing concentration of microparticles has been confirmed on the samples B–D as displayed in the images below. The average dimensions of the gelatin particles range somewhere between 40 and 250 microns. Certain irregularities have been noticed with respect to the shape of the microparticles; the geometry of the dried particles suggests a predominant spherical-like aspect; however, irregular shaped as well as particles agglomerations are clearly visible. Moreover, some fibrilar bridges are little distinguishable between the microparticles of the samples B–D at 100× magnification, but this aspect has been more detailed investigated at higher magnification as further described.

Collagen fibrils clearly distinguish on a collagen background in the control sample A. The samples loaded with gelatin present numerous fibrillar features connecting the microparticles. SEM does not allow an assessment on the nature of those structural features. However, some assumptions could be made, namely, the bridges may consist in collagen fibrils which formation has been favored by the presence of the gelatin particles. Normally, increasing the concentration of microbeads in the scaffolds leads to increasing the number of connection bridges. The higher gelatin loading of the collagen corresponds to a more difficult visualization of individual fibrillar linkages on a background made out of agglomerated microparticles. More continuous connections are visible, appearing like a “glue” layer between the beads. The inter-particles distance decreases with increasing gelatin content. When the magnification has been increased to 250×, the bridges between the microparticles have been even better observable. The average diameters of the observed fibrils range in a wide interval, between approximately 10 microns to even nano-level.

AFM analysis has been performed at a maximum magnification allowing for the screening of areas of 10 × 10 microns. This dimensional range presents an obvious limitation with respect to the main purpose of the study, namely, the investigation of the interfaces between gelatin and collagen. This observation/conclusion is based on the dimensions of the gelatin microparticles (micro-level) making impossible to distinguish, on such low areas, between collagen background, microparticles surface, and collagen fibrillar bridges. However, different areas have been analyzed and the obtained results are presented below.

**Figure 8:**
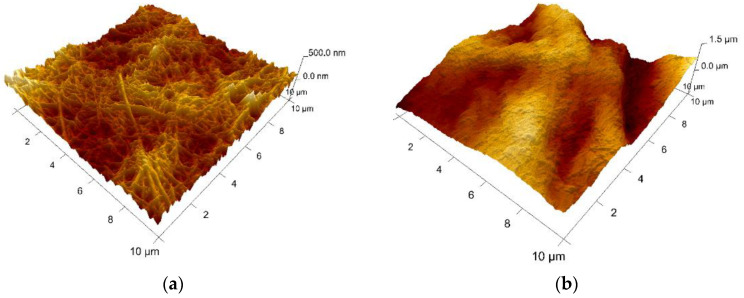

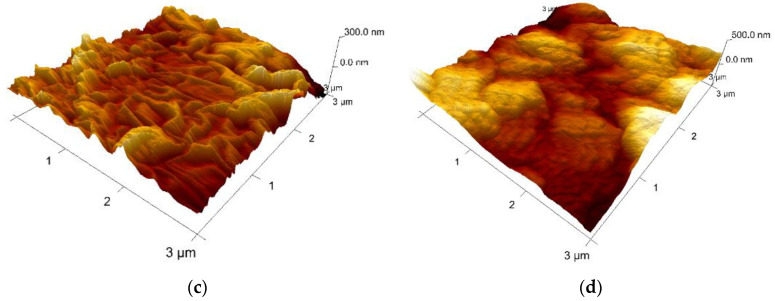
AFM 3D Images on the experimental samples collagen based loaded with gelatin microspheres: (**a**) sample A—0% gelatin (10 μm × 10 μm, z-range = 500 nm); (**b**) sample B—4% gelatin (10 μm × 10 μm, z-range = 1.5 µm); (**c**) sample C—21% gelatin (3 μm × 3 μm, z-range = 300 nm), and (**d**) sample D—41% gelatin (3 μm × 3 μm, z-range = 500 nm).

Collagen (sample A) appears as a network of fibrils with nano-sized diameters. Figure 8 shows the topography of sample A. Native fibrils and fibrous long spacing fibrils, which are specific of collagen, can be seen. Diameter of fiber is of about 95 nm. With respect to the microparticles-containing samples B–D, characterizations have been made on the surface of particles, as indicated in the microscopic images displayed for each sample. Different roughness has been observed, but, unfortunately, it cannot be concluded on interface interactions between particles and the collagen matrix.

Based on the experimental results, collagen loaded with gelatin microspheres present numerous fibrillar features connecting the microparticles and appear to be a good candidate material for the haemostatic agent.

## 5. Conclusions

Achieving haemostasis is a critical goal for intraoperative interventions, especially when it comes to cardiac surgery. Such interventions most commonly include mechanical haemostats (suturing lines, and anastomosis) and electrical cauterization of the tissue; however, the methods are in some cases deficient or inappropriate, given the different anatomic locations and specific procedures.

Haemostatic agents appear to be highly effective at decreasing the risk of bleeding during surgical procedures. Although some haemostatic agents were demonstrated to achieve haemostasis faster than others, most are able to control bleeding within <10 min. None of the current haemostatic agents meet the characteristics of an ideal haemostatic. The development for different novel broad-spectrum biomaterials and technologies in order to achieve all these criteria needed for a more efficacious haemostasis could be innovative. Further randomised controlled trials comparing the current haemostatic adjuncts would be beneficial to elucidate the efficacy and effectiveness of haemorrhage control.

The continuously growing number and diversity of haemostatic agents offers a variety of options to clinicians. It is, therefore, important to understand the mechanism of action and differences in order to select an optimal agent to achieve haemostasis and improve clinical outcomes for patients. Collaboration between emergency medical and surgical personnel, biomedical engineers, and material scientists is needed to identify and develop innovative solutions for novel haemostatic agents to revolutionize bleeding treatment approaches.

## Figures and Tables

**Table 1:** Classification of commercial haemostatic agents based on component used.

Commercial Haemostatic Agents		
I. Without Human or Bovine Component	Commercial name	Specifications/Composition
	Surgicel	Oxidized cellulose polymer
	Oxycel	
	Omnex	Synthetic tissue adhesive (Cyanoacrylates)
	Coseal	Combination of two polyethylene glycol polymers
	Arista	Microporous polysaccharide hemispheres
	Hem Con	Freeze-dried chitosan derived from shrimp shellContains chitosan
	Chitoseal	
	Celox	
	Recothrom	Recombinant thrombin (topical use)
II. With Bovine Component	BioGlue	Bovine albumin, and glutaraldehyde
	CoStasis	Bovine collagen, bovine thrombin, and calcium chloride mixed with autologous plasma at the time of surgery
	Thrombin	Bovine protein
	Avitene	Water-insoluble acid salts of bovine collagen
	Colgel	
	Helitene	
III. With Human Plasma Component	Tisseel	Human thrombin and fibrinogen, synthetic aprotinin
	Vivostat	Combination of platelets and autologous fibrin (prepare on site)
	Crosseal	Tranexamic acid, human plasma protein, thrombin
	Quixil	
IV. With Bovine and Human plasma component	Floseal	Human thrombin and bovine gelatine
	Beriplast	Human thrombin and fibrinogen, bovine aprotinin

## Data Availability

The data presented in this study are available on reasonable request from the corresponding author.
